# Mechanical properties of human oral mucosa tissues are site dependent: A combined biomechanical, histological and ultrastructural approach

**DOI:** 10.1002/cre2.305

**Published:** 2020-07-02

**Authors:** Joanne Jung Eun Choi, Johann Zwirner, Rishi Sanjay Ramani, Sunyoung Ma, Haizal Mohd Hussaini, John Neil Waddell, Niels Hammer

**Affiliations:** ^1^ Faculty of Dentistry Sir John Walsh Research Institute, University of Otago Dunedin New Zealand; ^2^ Department of Anatomy, School of Biomedical Sciences University of Otago Dunedin New Zealand; ^3^ Department of Clinical and Macroscopic Anatomy Medical University of Graz Graz Austria; ^4^ Department of Trauma, Orthopedic and Plastic Surgery University Hospital of Leipzig Leipzig Germany; ^5^ Fraunhofer IWU Dresden Germany

**Keywords:** Biomechanics, Elastic modulus, Oral mucosa, Tensile strength, Thiel‐embalming

## Abstract

**Aim:**

To investigate load‐deformation properties of Thiel‐embalmed human oral mucosa tissues and to compare three different anatomical regions in terms of mechanical, histological and ultrastructural characteristic with focus on the extracellular matrix.

**Materials and Methods:**

Thirty specimens from three different regions of the oral cavity: attached gingiva, buccal mucosa and the hard palate were harvested from two Thiel‐embalmed cadavers. Mechanical properties were obtained, combining strain evaluation and digital image correlation in a standardised approach. Elastic modulus, tensile strength, strain at maximum load and strain to failure were computed and analysed statistically. Subsamples were also analysed using scanning electron microscopy (SEM) and histological analysis.

**Results:**

The highest elastic modulus of 37.36 ± 17.4 MPa was found in the attached gingiva group, followed by the hard palate and buccal mucosa. The elastic moduli of attached gingiva differed significantly to the buccal mucosa (*p* = .01) and hard palate (*p* = .021). However, there was no difference in the elastic moduli between the buccal mucosa and hard palate (*p* > .22). The tensile strength of the tissue samples ranged from 1.54 ± 0.5MPa to 3.81 ± 0.9 MPa, with a significant difference between gingiva group and buccal mucosa or hard palate (*p* = .001). No difference was found in the mean tensile strength between the buccal mucosa and hard palate (*p* = .92). Ultrastructural imaging yielded a morphological basis for the various mechanical properties found intraorally; the attached gingiva showed unidirectional collagen fibre network whereas the buccal mucosa and hard palate showed multi‐directional network, which was more prone to tension failure and less elasticity.

**Conclusion:**

This is the first study assessing the various morphological‐mechanical relationships of intraoral soft tissues, utilising Thiel‐embalmed tissues. The findings of this study suggest that the tissues from different intraoral regions showed various morphological‐mechanical behaviour which was also confirmed under the SEM and in the histological analysis.

## 1 INTRODUCTION

Oral mucosa tissues are subjected to various levels of external forces especially during mastication (Boucher, 2004). Under these forces, oral soft tissues undergo elastic deformation to large extent (Kydd & Daly, 1982). The resiliency of these tissues is influenced by the thickness of the epithelium and the collagen fibre content of the submucosa which regulate mechanical properties (Scapino, 1967; Isobe et al., 2013).

In dentate patients, masticatory forces are dissipated through the long axis of the teeth. However, in edentulous patients wearing complete dentures, the masticatory forces are transmitted directly to the oral mucosa, causing an alteration of the underlying epithelium and connective tissue (Boucher, 2004). Within the connective tissue, a collapse of the collagen network has been observed in regions of stress (Sharma & Mirza, 1986). This alteration in collagen fibre networks could alter the resiliency of the tissues, which in turn would impact the dynamics of the tissue‐denture interface (Hayakawa et al., 1994). Any change in the mechanical properties of the mucosa could render it more prone to injuries and insults and decrease of the pressure pain threshold (PPT; Yoshida et al., 1999). In previous literature, the difference in PPT across different intraoral locations and on the mucosa has been attributed to the difference in mucosal thickness (McMillan, 1995). Most of the pain experienced when wearing removable dentures is a consequence of damage to the underlying tissue due to overloading (Szentpétery et al., 2005; Tanaka et al., 2004). However, there are still limited studies available on how the PPT varies between different intraoral sites.

The mechanical characteristics of the foundation mucosa can be described as the cushioning properties (resilience) that endow an ability to bear sustained or cyclic compression and tension (Kimoto et al., 2007). As one of the fundamental parameters to define material behaviour, the modulus of elasticity is the physical description of an object's tendency to be deformed proportionally to the applied force. The oral mucosa has been shown to be highly deformable under compression (Lytle, 1962) and the elastic modulus appears to vary over a broad range (Chen et al., 2015). Being a heterogeneous material, the mucosal instant stiffness results from both the solid matrix structure (e.g., epithelial layer, fibrous network, blood vessel, etc.) and the fluid components.

In order to evaluate the mechanical properties, previous studies used porcine oral mucosa tissues (Goktas et al., 2011; Lacoste‐Ferre et al., 2011). Several computational modelling/finite element analysis (FEA) studies attempted to simulate the pressure exerted from complete dentures onto the oral mucosa extending to the underlying alveolar bone (Chen et al., 2015). However, in those studies, the elastic modulus and tensile stress of the oral mucosa were incorrectly assumed to be all uniform in the mouth. This in turn affects the validity of the FEA simulations and the trends found from such studies.

Defining the mechanical characterisation of living human oral tissues has been difficult, due to the ethical issues and difficulties of sourcing tissue samples for testing. Fresh human cadaveric tissues are of extremely limited supply for biomechanical testing and they start to deteriorate rapidly with a potential risk for infection (Anderson, 2006). One study (Kydd & Mandley, 1967) which tested the soft tissues around the human teeth within an hour of harvesting them had only three subjects and the tissues were stored in Ringer's solution. Moreover, the age and gender of these cadavers were not listed. Two follow‐up studies (Kydd & Daly, 1982; Picton & Wills, 1978) measured the compressive stresses directly on the tissue in the participants using the pressure transducers. However, due to the lack of standardisation in the methodology, the reported compressive modulus data is questionable. Moreover, the compressive pressure can be only exerted until the subjects feel pain, and one's individual pain threshold varies.

The condition of post‐mortem storage such as embalming techniques should be taken into consideration when assessing the feasibility of using cadaveric tissue for measuring the mechanical properties. Chemicals such as glutaraldehyde or formaldehyde are commonly used for storing cadaveric specimen in order to prevent tissue‐deformation and preserve the histological structures of soft tissues (Comert et al., 2009; Whitehead & Savoia, 2008). However, it is also known to alter the biomechanical properties by extensive tissue cross‐linking (Hansen et al., 2009; Viidik & Lewin, 1966). On the other hand, Thiel embalming keeps the tissues pliant, flexible and with realistic colour and native articular joint mobility (Thiel, 1992; Ottone et al., 2016; Zwiner et al., 2019). This method has shown to maintain the tissue plasticity similar to the living tissues at the macroscopic level and therefore, it is well‐suited for anatomical and surgical training as well as biomechanics studies. Despite the numerous benefits, Thiel‐embalmed tissues have not been used in dentistry to characterise the biomechanical properties of oral mucosa. Understanding the stresses and strains produced upon mucosa with differing configurations of collagen fibres would allow us to predict the probability of plastic deformation of certain locations of mucosal tissue.

Therefore, the purpose of this study was to evaluate the modulus of elasticity and tensile properties of Thiel‐embalmed human oral mucosa tissues at different locations. The null hypothesis was that there was no difference in the elastic modulus and tensile properties of the oral mucosa tissues harvested from different intraoral sites.

## 2 MATERIALS AND METHODS

This method was heavily adapted from Zwirner et al. (2019). Prior to collecting the Thiel‐emalmed tissues, ethical approval was granted by the University of Otago Human Ethics Committee (Ref: H17/20). The Ngai Tāhu Research Consultation Committee was also informed and a Maori consultation obtained.

### 2.1 Retrieval and processing of human mucosa samples

**FIGURE 1:**
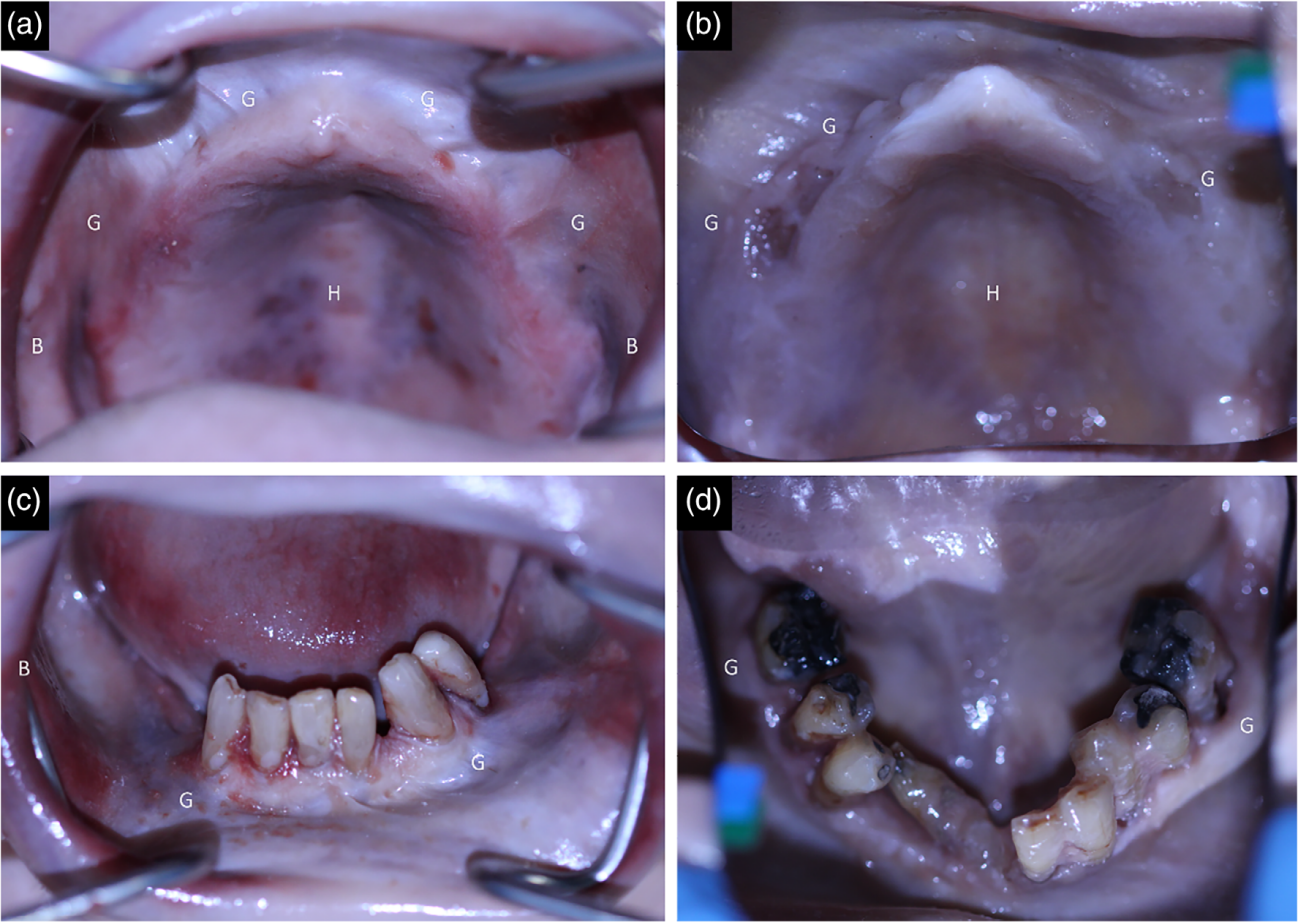
Intraoral photographs of donor 1 (a) maxilla and (c) mandible; donor 2 (b) maxilla (d) mandible. Note the natural preservation of color and texture of the tissues and marked areas where tissues were removed; G: Gingiva; H: Hard palate; B: buccal mucosa

**FIGURE 2:**
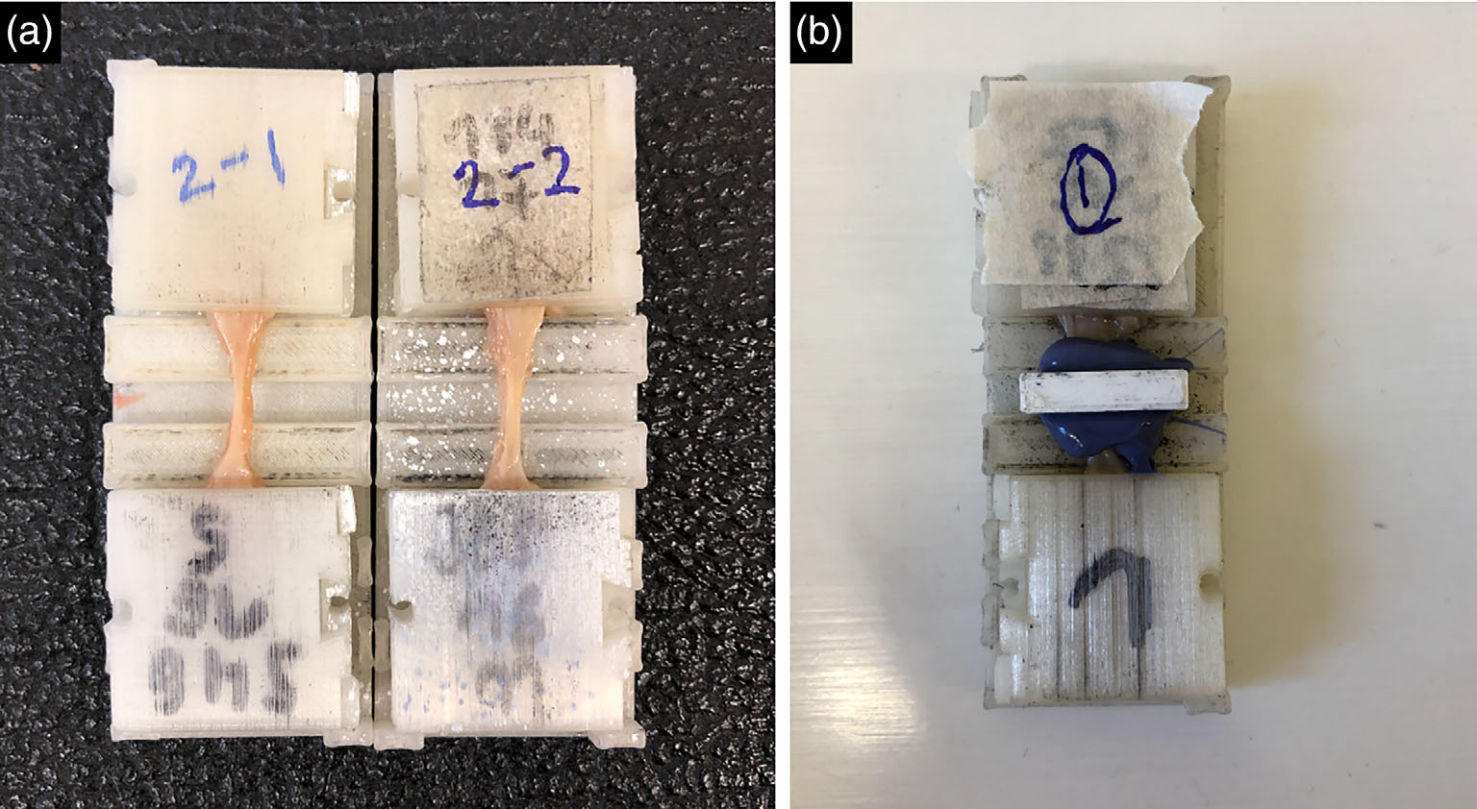
Images showing (a) the Thiel‐embalmed tissues clamped in 3D printed clamps to limit slippage during testing; (b) the mounting with the silicone material for cross‐sectional area calculation

A total of 30 oral mucosa samples from three different intraoral sites (buccal mucosa, hard palate and attached gingiva) were harvested from two Thiel‐embalmed cadavers at the Department of Anatomy, University of Otago, New Zealand (Figure 1) as per protocols described in Hammer et al. (2015) (Hammer et al., 2015). The age at the time of death for the Thiel‐embalmed cadavers was 69 and 81 years old. They were both partially dentate males (edentulous maxilla opposing partially dentate mandible). Commercially available premixed arterial infusion and tank fluids were used for the Thiel‐embalming according to the protocol by Zwirner et al. (2019). The samples were shaped to dumbbell shaped samples (Figure 2) following dimensions as per ISO 527‐2 standard (International Standard Organization, 1996).

### 2.2 Mechanical testing, scanning electron microscopy and histologic evaluation

After shaping, polysiloxane impression material (Medium bodied, Exahiflex, GC corporation, Tokyo, Japan) was used in the narrow portion (area of parallel measurement length; Figure 2). A commercial scanner (Perfection 7V750Pro; Seiko Epson Corporation, Suwa, Japan) was used to scan the casts of the cross sections at a 1,200‐dpi resolution, and the cross‐sections were calculated using the Measure 2.1d software (DatInf, Tubingen, Germany). A randomly distributed speckle pattern was created on the surface, creating random gray intensity distribution, necessary for the digital image correlation (DIC), being able to track facets and thus calculate in‐plane surface strain fields using this information. A perpendicular virtual extensometer was calculated and the nominal strain within two points was defined in the samples' testing length to make the test evaluation uniform. All Uniaxial tensile tests were performed at room temperature of 22°C using the same universal testing machine (Allround Table Top Z020; Zwick Roell, Ulm, Germany), with a Xforce P load cell of 2.5 kN with test Control II measurement electronics (all Zwick Roell). All specimens were clamped using speicifically designed 3D‐printed clamps to minimize slippage of the samples upon testing (Scholze et al., 2018; Zwirner et al., 2019). Prior to loading until failure, all samples were preconditioned with 20 load‐unload cycles at a force range of 0.5–2.0N. A cross‐head displacement rate of 20 mm/min and a sampling rate of 100 Hz were used for the load–displacement readings. The deformation of the specimen surface was recorded perpendicular to the surface by a DIC system with a resolution of 2.8 megapixels (Q400, Limess, Krefeld, Germany). The ISTRA 4D software (VRS 4.4.1.354; Dantec Dynamics, Ulm, Germany) was used for the evaluation of stress–strain data of the mechanical tests (Scholze et al., 2018; Zwirner et al., 2019).

In addition to the tensile testing, scanning electron microscopy (SEM) was performed on six Thiel‐embalmed samples (two per each group) using JEOL 6700 field emission SEM (JEOL, Peabody, MA). Coating of the samples was performed with a K575X sputter coater with a 5‐nm layer of gold palladium (Emitech Technologies, Kent, UK).

For the histologic evaluation, tissue samples were fixed with formalin and processed using a standard tissue processor (Citadel2000, Thermo Shandon, Runcorn, Cheshire, UK). The paraffin blocks were sectioned at 3–5 mm per section, mounted on standard glass slides, and stained with hematoxylin and eosin (H&E). Descriptive light microscopic examination (BX50, Olympus, Toyko, Japan) was completed and photomicrographs (DM5000B/DC50, Leica, Wetzlar, Germany) were taken at ×100 and ×200 magnifications.

### 2.3 Processing of data and statistical analysis

The statistical analysis was done using SPSS (IBM, Version 24). Normality of distribution was assessed using Shapiro–Wilk's test. Mean tensile and elastic modulus differences were compared between the samples harvested from three different locations by ANOVA. Subsequent post hoc tests were carried out to conduct multiple comparisons between the specimen groups with the significance of *p* < .05.

## 3 RESULTS

**FIGURE 3:**
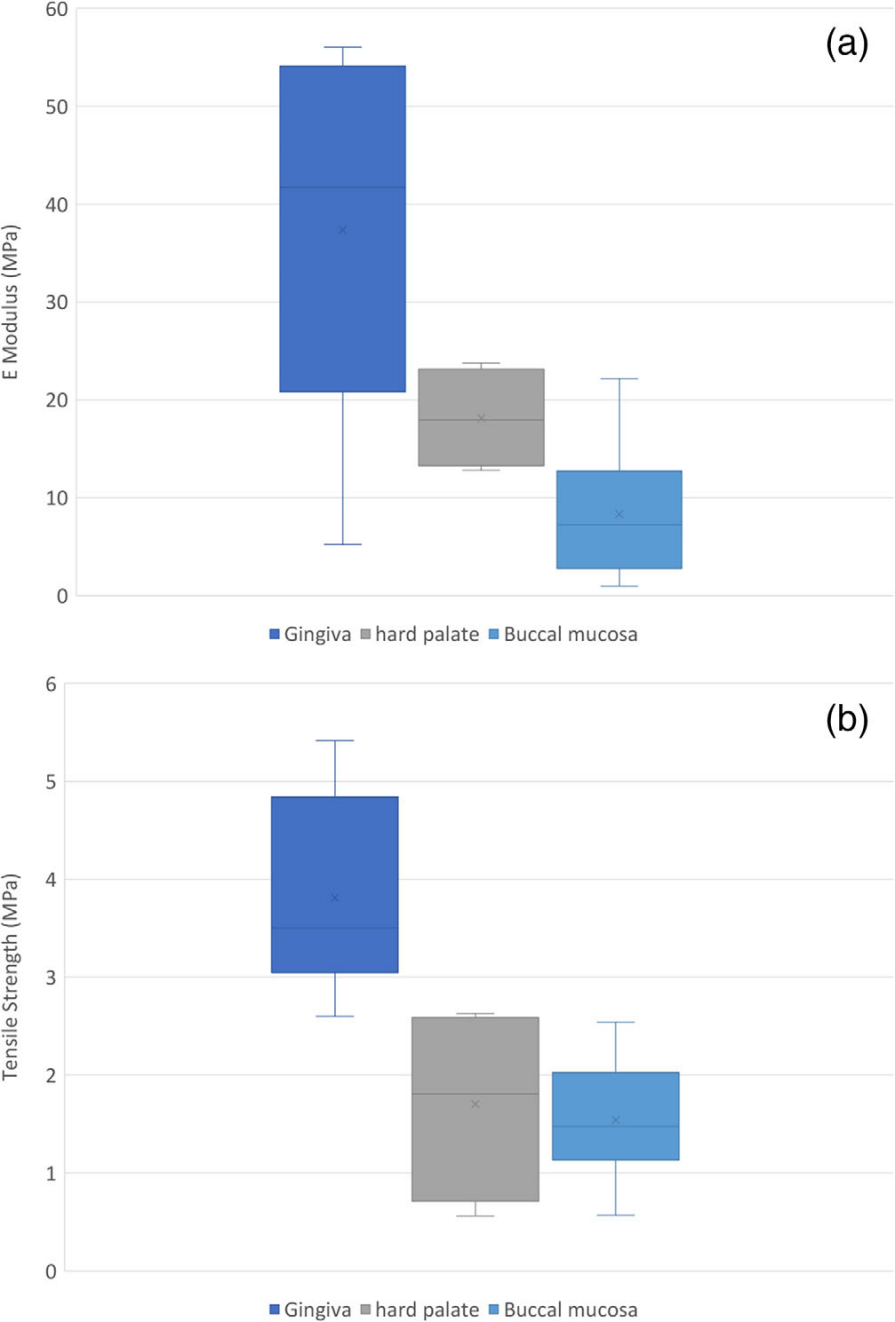
Graphs showing (a) elastic modulus and (b) tensile strength of all groups

**FIGURE 4:**
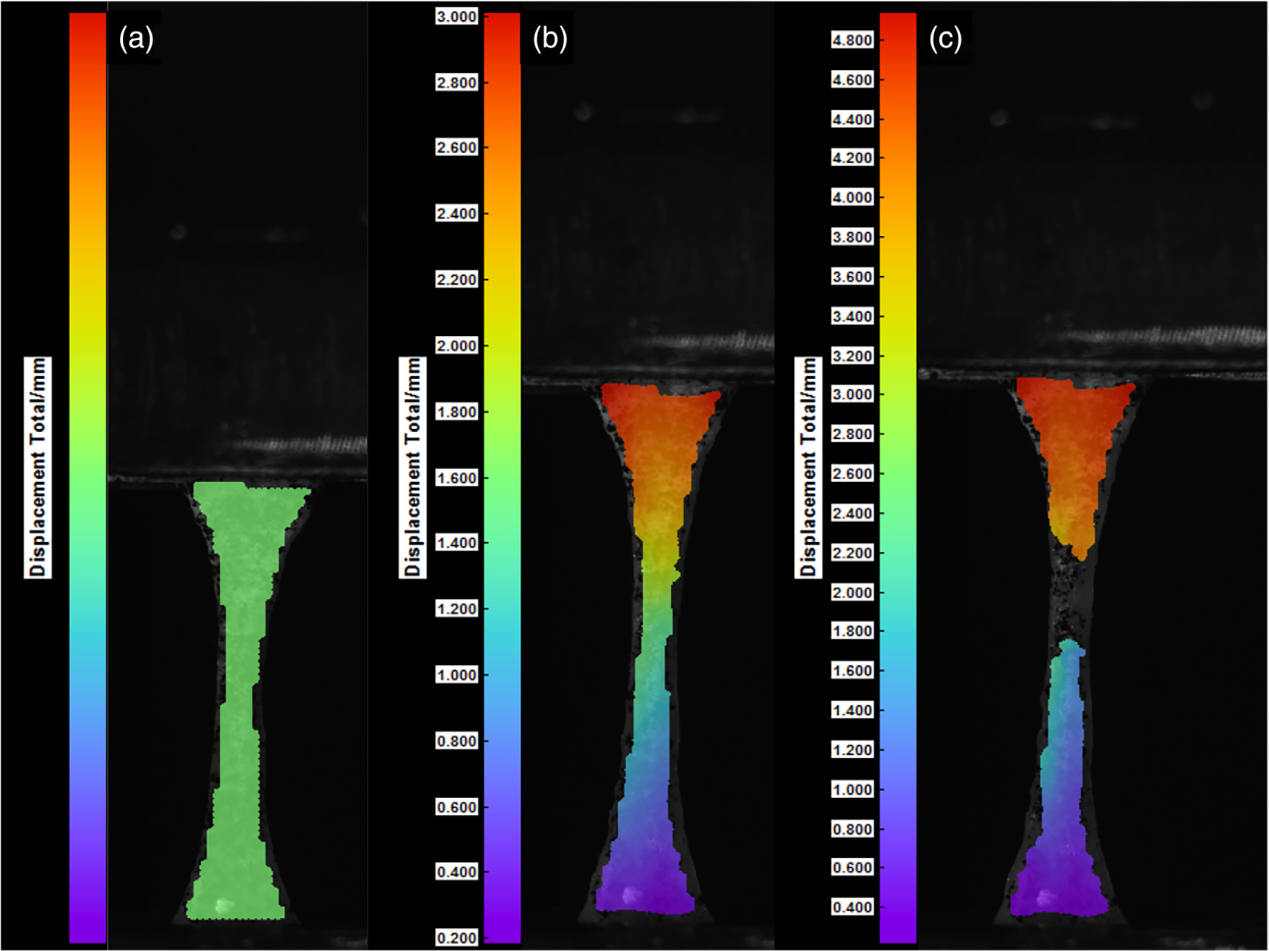
Digital image correlation (DIC) images showing a typical stress–strain curve and the appearance of the tested specimens at different points in time for oral mucosa under uniaxial loading conditions. (a) The DIC software shows no current displacement, indicated by the green color. After an increasing load is applied, the sample strains (indicated by a multicolored labeling in the DIC software. (b) Finally, a localized failure occurs in the area of the parallel specimen length, displayed by a discontinued color labeling in the DIC software (c)

The highest mean elastic modulus of 37.4 ± 17.4 MPa was found in the attached gingiva group, followed by the samples from the hard palate (18.1 ± 4.5 MPa) and buccal mucosa (8.3 ± 5.8 MPa) as shown in Table 1 and Figure 3a. The mean elastic moduli of attached gingiva was statistically different to the buccal mucosa and the hard palate groups (*p* = .01 and *p* = .021, respectively). However, there was no statistical difference between buccal mucosa and the hard palate (*p* = .22). The tensile strength of the tissue samples ranged from 1.5 ± 0.5 MPa to 3.8 ± 0.9 MPa (Figure 3a and Table 1), with a statistical significance observed between buccal mucosa and gingiva group or hard palate and gingiva (*p* = .001). However, there was no statistical significance found with the mean tensile strength between the buccal mucosa and hard palate (*p* = .92). There was also no statistical difference found in the two properties of the tissues samples between the two donors in terms of elastic modulus (*p* = .23) and tensile strength (*p* = .37). Digital image correlation (DIC) images showing a typical stress‐strain curve and the appearance of the tested specimens is shown in Figure 4.

**TABLE 1:** Elastic modulus (±*SD*) and tensile strength (±*SD*) of all groups

	E Modulus (MPa)	Tensile stress (MPa)
Gingiva	37.36 (±17.36)	3.81 (±0.94)
Hard palate	18.13 (±4.51)	1.70 (±0.87)
Buccal mucosa	8.33 (±5.78)	1.54 (±0.52)

**FIGURE 5:**
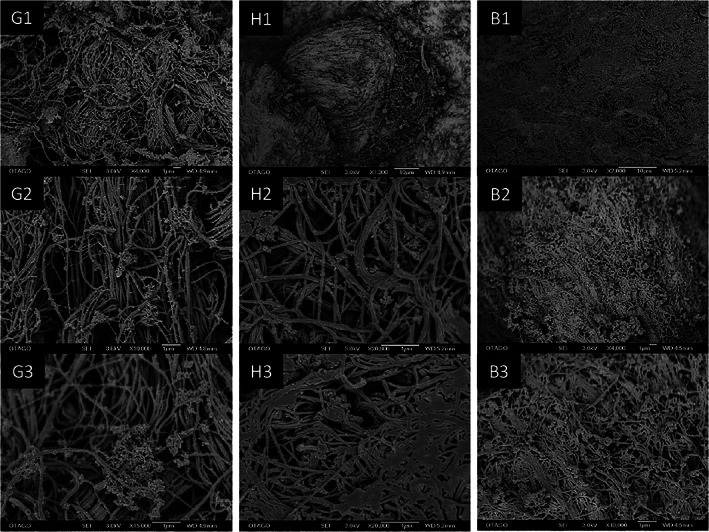
Scanning electron microscopy images of tissues samples harvested from different regions (G1‐3; gingiva; H1‐3; hard palate and B1‐3; buccal mucosa) at magnification of ×1,300 to ×20,000

The SEM images revealed different collagen fibre arrangements in the tissues harvested from the three different intraoral sites. Attached gingiva tissues showed predominantly unidirectional collagen fibre networks and unravelled elastin (Figure 5, G1–G3) compared to the hard palate and buccal mucosa tissues. Collagen coiling (bundles) and partial failures of the superficial collagen were found (Figure 5, G2 and G3) at higher magnification. The tissues harvested from the hard palate region showed denser fibres (Figure 5, H3) and reticular tissues at random direction. Moreover, tissues harvested from the palatal rugae area showed more myxoid characteristics (Figure 5, H1). Compared to the gingiva tissues, collagen fibres were found to be denser (thicker fibres) but less orientated (Figure 5, H2 and H3). Buccal mucosa tissues showed thinner bundles of fibres arranged in multiple directions (Figure 5, B1 and B3). At a higher magnification, the fibres appeared more packed (Figure 5, B3) in a random direction, which was also evident in the tissue samples harvested from gingiva (Figure 5, G3).

**FIGURE 6:**
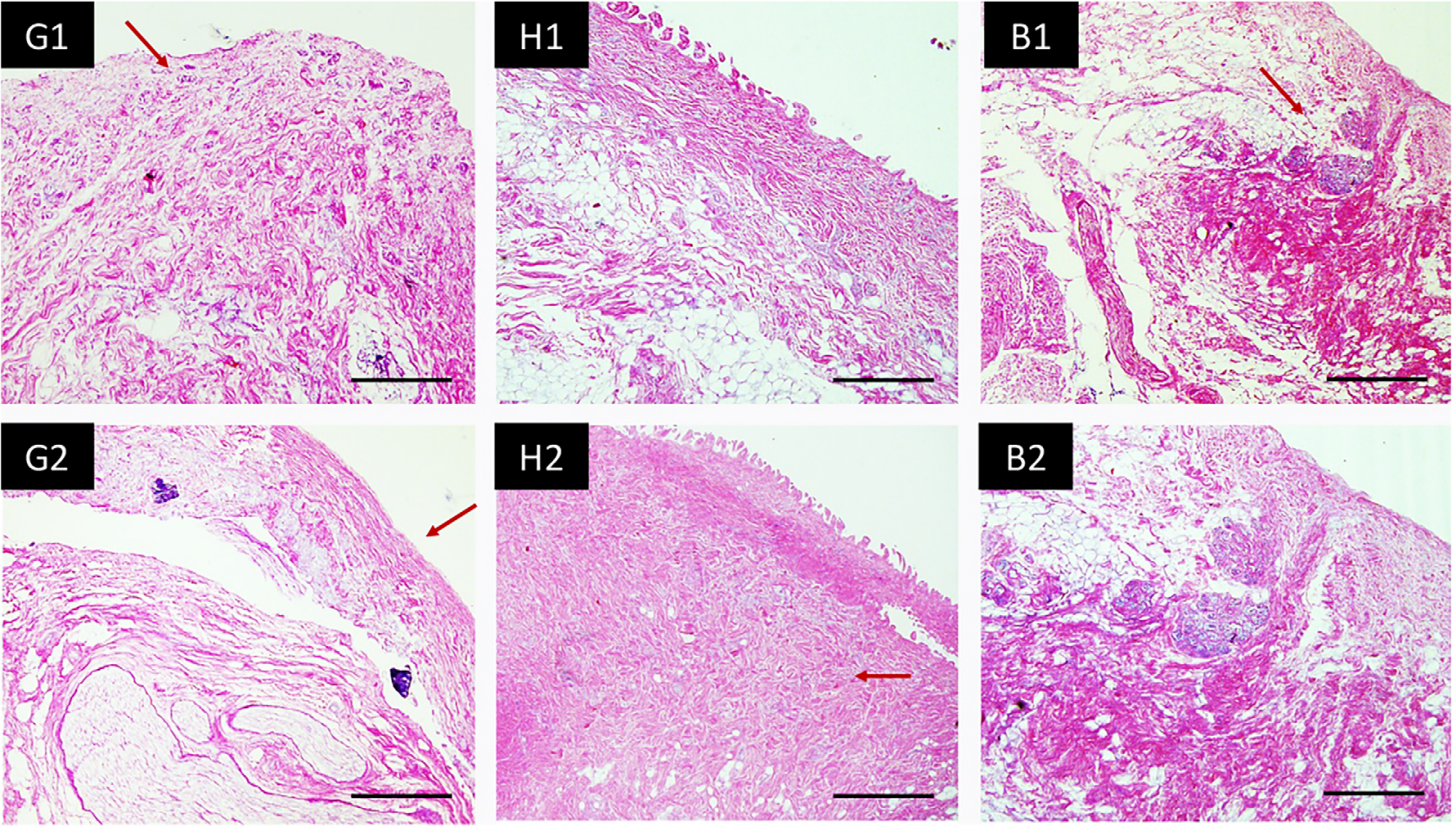
Hematoxylin and eosin images of tissue samples harvested from different intraoral regions; G: gingiva showing high content of elastic fibres (red arrow); H: hard palate showing densely packed collagen fibres (arrow) and B: buccal mucosa at 1: ×100 and 2: ×200 magnifications. Scale bar =50 μm

H&E stained sections of the tissues also revealed that gingival tissues have densely oriented collagen tissues arranged perpendicular to the loading (Figure 6, G2). High content of elastic fibres (curliness found in Figure 6, G1) and the lowest fat content was found in gingiva tissue samples compared to the others. At higher magnifications (×200), the gingival tissues showed more anisotropic properties, whereas buccal mucosa tissues showed isotropic properties (Figure 6, B1 and B2). In the buccal mucosa tissues, Type 1 collagen bundles were visible, but it had the lowest collagen content and the fibres were not perpendicularly arranged to the loading. Some empty areas were visible, most likely due to the chemical treatment of the tissues. Lastly the hard palate tissue showed intermediate characteristics between the gingiva and buccal mucosa tissues; showing densely packed collagen fibres distributed in a more multi direction (Figure 6, H1 and H2) compared to the gingiva tissues (Figure 6, G1 and G2).

## 4 DISCUSSION

This is the first study which showed the feasibility of the using Thiel‐embalmed tissues to investigate the human oral mucosa to which a direct comparison could be made between tissues from different intraoral regions. The null hypothesis stating that there would be no difference in the elastic modulus and tensile properties of the oral mucosa tissues harvested from different intraoral sites was rejected as the findings of this study suggest that the mucosa tissues from different intraoral region have different mechanical behaviours.

Oral soft tissues are complex biologic systems with the components of their extracellular matrix responding differentially to physiologic stresses. The tissues are subjected to a wide variety of mechanical forces, including hydrodynamic forces, compression, tension, friction and shear generated during saliva flow, mastication and speech (Boucher, 2004). Oral mucosa consists of different groups of fibres, depending on their locations in the oral cavity to withstand different ranges of masticatory loads. For example, attached gingiva is composed of connective tissues with a thickness of 0.15–0.35 mm, consisting of blood vessels, collagen, osytalan and eulanin fibres, which range up to 50% of the total tissue volume (Zwirner et al., 2019). It is known that the existence of blood vessels in the tissues and the hemodynamic pressure that these vessels exert affects the biomechanical response. However, there is a lack of evidence describing the biomechanical variation that occurs regionally and the relationship between distinct mechanical zones and tissue structure. The biomechanical response of the oral mucosa to the occlusal loads is also not clear. Tsaira et al. (2016) hypothesised that under tension, the wavy configuration of the fibres will be responsible for the load transmission from the tooth to the neighbouring alveolar bone via a gradual unfolding of these fibres (Tsaira et al., 2016). This hypothesis was evident in the findings of the present study as under tension, the gingiva tissues showed the highest tensile strength and elastic modulus. SEM and H&E images supported this finding since the gingiva tissue had more unidirectional distribution of the collagen fibres, perpendicular to the loading, thus dissipating the load effectively. The highest mechanical properties found in the attached gingiva group showed that the fibres can withstand higher loads compared to the tissues from the hard palate and buccal mucosa region, which had more random distribution of the fibres, creating a weaker structure under masticatory loading.

On the other hand, buccal mucosa has shown the presence of elastic fibres which decrease in density in areas adjacent to gingival connective tissue (Bourke et al., 2000). When observed, the buccal mucosal connective tissue displayed a loose fibre network sparsely populated with collagenous fibres but also possessed abundant elastic fibres (Meyer & Gerson, 1964), which was also evident in the current study. Buccal mucosa, which has a reduced exposure to abrasive forces, is relatively loosely attached to the underlying bone and possess a non‐keratinizing epithelium. This may be the reason for the lowest mechanical properties found in the buccal mucosa tissues.

Despite the attention given to the mechanical properties of oral tissues from different regions, there is only one previous study by Goktas et al. (2011) which used porcine oral mucosa tissues (Goktas et al., 2011). Their study also revealed that there was a significant regional variation directly related to unique structural and functional characteristics of different tissues. The tensile properties of the porcine oral mucosa tissues were within the range of the data obtained from the current study; 1.06–3.94 MPa for the tensile strength of the porcine tissues, whereas 1.00–3.8 MPa was the range found for the tensile strength of the human oral mucosa tissues found from the current study. The ultimate tensile strength of the buccal attached gingiva (3.94 MPa) was similar to that of the lingual attached gingiva, and this strength value was significantly higher than all other regions of oral mucosa tested. Similarity in the results seen in the current study supports the previous notion that porcine tissues may also be used as the substitute tissues for the biomechanical studies. However, the elastic modulus of porcine mucosa was found to be significantly lower compared to that of the human oral mucosa tissues.

The difference between Goktas et al. (2011) and current data can be due to the difference in the tissue compositions between the human and porcine mucosa tissues (Goktas et al., 2011). Human tissues typically display an increase in collagen and non‐collagenous protein content, which may have contributed to the higher and wide range of the elastic moduli found in oral tissues. Moreover, in Goktas et al.'s (2011) study, although tissues were harvested from different regions, they were still very close to each other and limited to the region around the teeth, due to the smaller oral cavity of pigs, whereas the tissues harvested in the current study were from a wider area, and sections that were well demarcated. The tissue properties from the hard palate was not available from Goktas et al.'s (2011) study, limiting the direct comparison of the mechanical properties. Lacoste‐Ferre et al. (2011) also used porcine oral mucosa to characterise the viscoelastic behaviours of the tissues, however, only attached gingiva was studied and the mechanical properties of the tissues, age of the pigs when the tissue samples were harvested and number of specimens were not specified (Lacoste‐Ferre et al., 2011). This lack of information meant that it was difficult to make any direct comparisons with the previous study (Goktas et al., 2011) and the findings from the current study.

At the early stage of exploring the stress–stain relationship of the mucosa, the experimental reports showed a wide range of possible compressive elastic moduli from 0.06 to 8.89 MPa when using a ‘dead’ weight or an instant load (Goktas et al., 2011; Kydd & Mandley, 1967; Tomlin & Wilson, 1968; Inoue et al., 1985). Meanwhile, there were several under tension than compression, showing elastic moduli ranging from 0.91 to 11.12 MPa (Kydd & Mandley, 1967), which correlates with our study, although the values are low. The modulus elasticity and viscous coefficient in a parallel model measured 1.1 and 250 MPa and in a serial model, they measured 1.2 and 18 MPa (Tanaka, 1973), respectively, which is similar to the finding of the current study. Different values have also been found in Hayakawa et al.'s (1994) study (Hayakawa et al., 1994), 0.36–0.59 MPa and 667–689 MPa for elements of a parallel model and 1.41 MPa and 56–63 MPa for elements of a serial model. Inoue et al. (1985) measured the modulus of elasticity of mucosa with ultrasound and reported the data is in the range of 0.91–5.93 MPa (Inoue et al., 1985). There is a broad agreement with this among other mechanical measurements taken of 0.37–5.80 MPa (Tanaka, 1973), 0.41–2.67 MPa (Nakashima, 1975), 0.66–4.36 MPa (Inoue et al., 1985) and 2.75–5.03 MPa (Jozefowicz, 1970).

Such broad range of elastic modulus and mechanical properties resulted in a difficulty of adopting computer modelling or FEA in dental prosthodontic research (Chen et al., 2015). Previous studies attempted to model the linear mucosal elasticity and the associated responses with dental prostheses, (e.g., complete and partial dentures, intraradicular posts, bridges and implants) with various models – including linear elastic, biphasic, multiphastic elastic and hyperelastic models. A broad range of elastic modulus values have been adopted in research, often by assumption. Initially owing to a lack of sufficient experiment data, the human skin (19.6 MPa) for being another typically soft tissue (Davy et al., 1981). Another two elastic modulus values 10 and 5 MPa were first reported in non‐English journals (Chen et al., 2015). Both such assumptions gained considerable acceptance. To simulate the effects of different mucosa resiliency to compression, elastic moduli of 340 and 680 MPa were assumed for the hard and medium mucosa, respectively, compared to the soft mucosa (1 MPa). Several FEA studies adopted the values between 1 and 5 MPa (Bourke et al., 2000; Kydd & Mandley, 1967) for simulations (Chen et al., 2015). All these linear elastic models from the literature assumed linearity with homogeneity and isotropy of the mucosa, although it has been anatomically demonstrated as a heterogeneous and anisotropic composite material, which was evident in the current study's SEM and H&E analysis. The elastic modulus values used are also around 10–20 times higher than the elastic modulus values obtained from the current study with Thiel‐embalmed tissues. At the other extreme, a very low elastic modulus of 0.1 MPa was also assumed and so was 0.68 MPa in other studies (Verri et al., 2011). This over‐simplification of the mechanics and model and the use of the values obtained from non‐living tissue may be useful for computational efficiency, but the wide range and inaccurate input value can exaggerate the output values via FEA, leading to false or inaccurate predictions of the study model and mucosa's responses to the dental prosthesis.

Although limited by the number of available human tissue samples and inherent inter‐specimen variability, Thiel‐preserved oral mucosa used in the current study showed the feasibility to efficiently characterise the mechanical properties to improve the accuracy of future simulation and computational modelling. This study has several other limitations, mostly regarding the human Thiel‐embalmed specimen testing. Although the differences in mechanical properties of the tissues harvested from two cadavers were found not to be statistically significant, there is still a need for future studies to categorise the age, gender, and lifestyle of the donors to minimise any variations. A follow‐up study with an increase in tissue sample number and from different intraoral sites is also recommended. An important aspect of Thiel‐embalming is that the fixatives cause extensive decellularisation of the epithelium and therefore, the epithelium and its keratinization are not identifiable in Thiel‐embalmed tissues. Moreover, fresh and unembalmed tissues are the gold standard since Thiel embalming causes (partly irreversible) changes in the mechanical properties of the tissues, especially if the tissues have little extracellular matrix and are rich in cells (Hammer et al., 2015). Despite these limitations, the extraepithelial matrix biomechanics are largely maintained, which can still give valid interpretations for mechanical properties (Zwiner et al., 2019).

Since the current study had a limited number of Thiel‐embalmed tissue samples, the mucosa thickness was kept consistent (following dimension according to the ISO standard). However, Goktas et al. (2011) reported that the modulus of elasticity varies considerably in the same subject and between individuals, even when testing porcine tissues (Goktas et al., 2011). Therefore, future studies with a higher number of tissue samples from an increased number of donors would be useful to investigate the correlation of mechanical properties of the thickness of mucosa and the relationship between the underlying residual ridge.

This study evaluated the mechanical variation as a function of three different tissue locations. A more detailed knowledge of the properties of these tissues, in particular functional and more specific regional variations, will provide an important baseline for the development of improved materials to repair or regenerate these soft tissues and simulation models. Moreover, understanding the mechanical characteristics of oral mucosa and tissue structures will enhance our ability to develop better man‐made materials and structures, particularly materials and systems that exhibit counter‐intuitive properties. Studying the biomechanical behaviour of the oral mucosa is essential to improve the denture‐bearing foundations for complete and partially edentulous patients, by better managing traumatized tissues and giving instructions to patients regarding the time required for tissues to recover, after applying occlusal loads.

This information could be also used to identify areas where using denture liners pre‐emptively would provide protection to tissue with a lowered tensile strength. A quantitative mechanical analysis of human oral tissues is essential for both materials development and clinical perspectives, if new materials are to be developed that mimic the behaviour of the natural tissues.

## 5 CONCLUSION

This is the first study which showed the feasibility of the use of Thiel‐embalmed tissues to investigate the human oral mucosa to which a direct comparison could be made between tissues from different intraoral regions and to elucidate the morphological‐mechanical relationship. The findings of this study suggest that the tissues from different intraoral region showed different mechanical behaviour, which was also revealed under SEM and in the histological analysis.

## CONFLICT OF INTEREST

The authors declare no conflict of interest.
